# The politics and ethics of hospital infection prevention and control: a qualitative case study of senior clinicians’ perceptions of professional and cultural factors that influence doctors’ attitudes and practices in a large Australian hospital

**DOI:** 10.1186/s12913-019-4044-y

**Published:** 2019-04-02

**Authors:** Gwendolyn L. Gilbert, Ian Kerridge

**Affiliations:** 1 Sydney Health Ethics, University of Sydney, Level 1, Building 1, Medical Foundation Building, 92/94 Parramatta Rd, Camperdown, NSW 2050 Australia; 2 Marie Bashir Institute for Infectious Diseases and Biosecurity, Westmead Institute for Medical Research, 176 Hawkesbury Rd, Westmead, NSW 2145 Australia; 3 Department of Haematology, Royal North Shore Hospital, Reserve Rd, St Leonards, NSW 2065 Australia

**Keywords:** Infection prevention, Healthcare-associated infections, Clinical autonomy, Leadership, Accountability, Unprofessional behaviour

## Abstract

**Background:**

Hospital infection prevention and control (IPC) programs are designed to minimise rates of preventable healthcare-associated infection (HAI) and acquisition of multidrug resistant organisms, which are among the commonest adverse effects of hospitalisation. Failures of hospital IPC in recent years have led to nosocomial and community outbreaks of emerging infections, causing preventable deaths and social disruption. Therefore, effective IPC programs are essential, but can be difficult to sustain in busy clinical environments. Healthcare workers’ adherence to routine IPC practices is often suboptimal, but there is evidence that doctors, as a group, are consistently less compliant than nurses. This is significant because doctors’ behaviours disproportionately influence those of other staff and their peripatetic practice provides more opportunities for pathogen transmission. A better understanding of what drives doctors’ IPC practices will contribute to development of new strategies to improve IPC, overall.

**Methods:**

This qualitative case study involved in-depth interviews with senior clinicians and clinician-managers/directors (16 doctors and 10 nurses) from a broad range of specialties, in a large Australian tertiary hospital, to explore their perceptions of professional and cultural factors that influence doctors’ IPC practices, using thematic analysis of data.

**Results:**

Professional/clinical autonomy; leadership and role modelling; uncertainty about the importance of HAIs and doctors’ responsibilities for preventing them; and lack of clarity about senior consultants’ obligations emerged as major themes. Participants described marked variation in practices between individual doctors, influenced by, inter alia, doctors’ own assessment of patients’ infection risk and their beliefs about the efficacy of IPC policies. Participants believed that most doctors recognise the significance of HAIs and choose to [mostly] observe organisational IPC policies, but a minority show apparent contempt for accepted rules, disrespect for colleagues who adhere to, or are expected to enforce, them and indifference to patients whose care is compromised.

**Conclusions:**

Failure of healthcare and professional organisations to address doctors’ poor IPC practices and unprofessional behaviour, more generally, threatens patient safety and staff morale and undermines efforts to minimise the risks of dangerous nosocomial infection.

## Background

In high income countries, an estimated 4–8% of hospital inpatients develop healthcare-associated infections (HAIs) [1] and nosocomial transmission of multidrug resistant organisms (MRDO) is a major contributor to antimicrobial resistance (AMR) and its associated healthcare costs [2, 3]. It is estimated that 35–55%, or more, of HAIs are preventable [4, 5], although rates are highly variable, depending on how effectively IPC measures are implemented [6, 7]. Failures of routine hospital infection prevention and control (IPC) practices, in high income countries during this century, have resulted in devastating nosocomial outbreaks of exotic or emerging infections, such as severe acute respiratory syndrome (SARS) in Toronto, in 2003 [8] and Middle East respiratory syndrome (MERS) in Seoul in 2015 [9], causing preventable deaths and massive social and economic disruption.

Hand hygiene is the most obvious, easily audited and, arguably, the most effective IPC practice [10, 11], Its efficacy has been recognised since at least the mid-nineteenth century, with numerous studies showing that significant reductions in pathogen transmission and HAI rates are temporally associated with improved hand hygiene compliance [12]. Nevertheless, the use of hand hygiene as a surrogate for IPC quality and the moral status of noncompliance have been questioned, largely as a consequence of ongoing controversy about auditing methods and plausible compliance targets [13–15]. Ethical considerations have particular salience in light of numerous studies reporting lower than average compliance with IPC policies among doctors, compared with other health professionals, albeit with wide variation [16–18]. Doctors’ attitudes and behaviours are important, because they disproportionately influence those of other hospital staff and doctors often overestimate their own knowledge and compliance [19, 20]. Yet their peripatetic clinical practice provides numerous opportunities to transmit pathogens [21] and to be pathogen “super-spreaders” [22, 23].

Doctors retain considerable professional autonomy and power, despite repeated challenges from increased regulation, other health professions, evidence-based medicine and consumerism [24–26]. Despite recent attempts to redefine “medical professionalism”, a universally agreed definition remains elusive; but all versions include common commitments to e.g. patient welfare; maintenance of knowledge and skills; and securing public trust through professional self-regulation and avoidance of conflicts of interest [27–29]. How doctors interpret these commitments depends on how they perceive their professional identity [30]. In practice, their attitudes and practices are complex and sometimes perplexing. Assuming that patient welfare *is* doctors’ highest priority [31], one may reasonably ask why some would expose their patients to preventable infection risks by failing to observe well-established IPC rules [30, 32]?

Previous qualitative and mixed methods studies of factors that affect adherence to IPC practices have generally involved mixed groups of healthcare workers and/or focused on particular institutional settings, such as intensive care units. While these studies have identified factors that contribute to IPC practices including: self-protection, role modelling, belief (or not) in the effectiveness of IPC, knowledge, communication and workload [11, 20, 33, 34] they have not, for the most part, explained why these factors are so influential. The aim of this study was to explore *what* factors affect *doctors’* IPC practices and, more specifically, *why* they are so influential. It took the form of in-depth conversations between researchers and participants, all of whom were senior clinical leaders and or clinician-director/managers with many years’ experience. Our expectation was that the perspectives of both “insiders” [senior doctors] and more objective “outsiders” [senior nurses] would provide new insights to inform strategies to raise the priority of IPC within the medical community and limit harm from HAIs and AMR more effectively.

Our research question was:

What professional and cultural factors influence doctors’ attitudes to and practice of infection prevention and control?

## Methods

### Research team

#### Personal characteristics

Both researchers are senior physicians, one female, one male. Our special interest in hospital IPC stems from longstanding experience in caring for patients who are at high risk, have suffered and, in some cases, died, from preventable HAIs and/or have experienced the uncertainty and fear associated with being colonised by a MDRO.

#### Relationship with participants

Both of us were employed, for many years before or during this study, at the hospital where it was conducted. Most of the participants were known to at least one of us, as colleagues, but with few exceptions, we had not worked closely with them.

### Study design

#### Theoretical framework

Our aim was to build a rich portrait of doctors’ attitudes to and practice of IPC, in one large Australian tertiary hospital, by means of in-depth interviews with experienced senior doctors and nurses. Together with them, we hoped to formulate new theories to better explain the reasons behind doctors’ IPC practices, in order to develop more effective and generalisable strategies to improve them. We used a thematic analysis approach to data analysis [35].

#### Participant selection

Participants were senior doctors (i.e. medical practitioners) and nurses with varied clinical and/or management responsibilities, who were purposively selected as being likely to have a broad perspective on the attitudes, beliefs and practices of the hospital’s medical staff across age-groups and specialties. Invitations were sent by email, explaining the purpose of the study. Thirty-two potential participants - 22 doctors and 10 nurses - were contacted sequentially. All of the nurses and 16 doctors agreed to participate; two doctors were unavailable, one refused and three did not respond. Sixteen participants were facility/divisional directors or unit managers, 10 were specialist consultants; they had had 10–40 years’ professional experience and most had been employed in the hospital for more than 10 years. Doctors were either full-time staff specialists [SS] or visiting medical officers (VMOs) contracted on a sessional basis. Nurses were all full-time employees. Participant characteristics are summarised in Table 1.

**Table 1:** Characteristics of participants

	Doctors [*N* = 16]: 11 staff specialists [SS]; 5 visiting medical officers [VMO]		Nurses [*N* = 10]	
Gender M: F	10:		3:7	
Seniority	Divisional director	6	Hospital/divisional director or deputy; facility manager	6
	Departmental/unit director	4	Nursing unit manager	2
	Other senior medical consultant	6	Clinical nurse consultant	2
Specialty	Medical 6; surgical 4; other 6^a^	16	Medical 5; surgical 3; other 2^b^	10

^a^Other doctors’ specialties: intensive care [2]; emergency medicine [2]; anaesthetics [1]; obstetrics/gynaecology [1]

^b^Other nurses’ specialties: intensive care, infection prevention and control

#### Setting

The setting was a ~ 950-bed university teaching hospital, serving a population of about 0.9 million in Sydney and providing a broad range of tertiary specialist services to more than 90,000 inpatients, annually. The study was conducted at a single site to enable a rich and detailed understanding of the influences on doctors IPC practices and to avoid potential confounding effects of different policies, patient populations or physical environments.

#### Data collection

Semi-structured interviews, lasting 27–77 min, were conducted by one researcher. They took the form of frank conversations between colleagues. Most were conducted in the participant’s - or occasionally the interviewer’s - office with no-one else present. Interviews were recorded and digitally transcribed by a professional transcription service, with participants’ informed consent. Examples of questions used to prompt discussion are shown in Table 2. Interview transcripts were checked for accuracy by one researcher.

**Table 2:** Interview topics/questions used to prompt discussion with participants

1. In your view, to what extent do doctors generally regard healthcare associated infections or colonisation with multidrug resistant pathogens as significant problems in this hospital?	
a. Do they/you believe that some/most could be prevented? b. What would it take to reduce the risks?	
2. There is evidence from audits and published research that doctors often comply less consistently than nurses with infection prevention and control measures.	
a. Is this consistent with your experience and, if so, why do you think it is so? b. Do you believe that it adversely affects patients’ outcomes?	
3. What changes, if any, would you make in hospital infection prevention and control policies or their implementation to:	
a. make them more acceptable to doctors, b. increase doctors’ adherence to them and/or c. achieve their goals of reducing risks to patients?	

### Analysis and findings

#### Data analysis

Both authors reviewed the transcripts, coded them manually and analysed them thematically [35], to identify themes and subthemes relevant to the research question. After initial analysis, transcripts and emergent themes/subthemes were reviewed, iteratively, and modified after further discussion. Recruitment ceased when data saturation had been reached.

### Ethics

Participants gave written informed consent to interviews being recorded and transcribed. They were assured that their comments would be confidential and quoted, if at all, only after removal of identifying information. In describing our findings, we identified participants’ professions and roles, broadly, to preserve anonymity, as *nursing* director (ND), manager (NM) or consultant (NC); or *medical* director (MD) or consultant (MC).

The relevant Local Health District human research ethics committee approved the study.

## Results

Four major themes emerged from the data and, within Theme 1, four subthemes were identified. These themes and subthemes are outlined in Table 3. Theme 1 is the subject of this paper. Other themes/subthemes will be discussed, in detail, elsewhere.

**Table 3:** Major themes identified by analysis of interview transcripts – factor affecting doctors’ attitudes to and practice of IPC

1. Characteristics of doctors, medical culture and medical professionalism	
i. Clinical independence/autonomy ii. Leadership and role modelling iii. Doctors’ accountability for healthcare associated infections iv. Consultants’ professional obligations	
2. Interprofessional factors	
3. Organisational factors	
4. Factors relating to IPC policies and/or their implementation	

### Clinical independence/autonomy

You can’t tell doctors what to do (medical director [MD]1).

Doctors’ highly valued clinical autonomy was seen as one of the most important determinants of IPC practices. Participants described doctors, generally, as self-motivated and averse to being told what to do, particularly as they become more senior. This was attributed to: the types of people who become doctors; doctors’ perception that others expect them to be confident and decisive; and their tendency to rely on clinical judgment and experience, rather than “rules”. However, participants described differences in how doctors enact clinical autonomy in their IPC practices. Those of some individual doctors and units - representing such varied specialties as transplantation surgery, haematology, general surgery and neurosurgery - were described, by participants, as exemplary. A unit director (MD4) recounted his own unit’s practice, of having a junior doctor audit the team’s hand hygiene compliance and present the results at weekly meetings, and his pride in the resulting improvement. At the other extreme, several participants – independently - cited examples of units in which IPC standards were notoriously poor and HAI rates (reputedly) excessive; they also described individual consultants who had to be reminded, repeatedly, about basic IPC precautions and who vehemently demurred, refused or tried to avoid situations where they may be asked, to comply:

From a political point of view, we (intensive care unit [ICU] clinicians) are in a bind. We encourage outside (surgical) teams to see their high-risk patients daily, (but) they're saying “I don't want to go to ICU, they always give me a hard time about washing my hands or taking my jacket off”. So, we're trying to make sure that they have easy access to the patients, but in such a way that the whole process is smooth. For some clinicians, hand hygiene presents *an objectionable obstacle*. (emphasis added) (MD3)

Several (doctor) participants thought their colleagues’ objections, to IPC “rules” that they perceived to be inflexible, inappropriately applied or imposed by outsiders, were legitimate. For example, patients being placed in contact isolation, when the risk of cross-infection was minimal, caused increased pressure on already over-burdened junior doctors and potentially endangered other patients, because of misplaced priorities.

The infection control nursing staff don’t understand the pressures that medical staff, particularly junior medical staff, are under. Some of the attitudes to infection control appear to be given in isolation without really an understanding of how they might be integrated into all the various things that have to be done by residents and senior doctors and nurses. (medical consultant [MC]1)

A bit of an irritant is if I’m asked to take precautions that I don’t think are appropriate (when) nursing staff have instituted a policy (and) gone a bit too far. (For example) a patient who has minor neutropenia and a (mild) fever that you’re keeping in just in case.... they’re given priority, put in a single room and everybody’s in gloves and gowns; and they’ve got other people out in the ward coughing and hacking, perhaps harbouring something much more significant. (MD4)

Several medical directors attributed doctors’ poor IPC practices to ignorance, although on-line IPC training and attendance at education sessions was supposedly mandatory.

There’s a poor understanding of what’s required, even though you’re meant to do mandatory training. But getting people to turn up - I honestly don’t know. (MD5)

In a VMO model you’re funded to do your clinic session or your (operating) list but not education (or) teaching. We should pay them for participating in education and training. (MD6).

Apparent adherence to IPC principles sometimes reflected tradition or habituated behaviour, rather than knowledge of organisational policies. A surgeon contrasted his colleagues’ almost ritualistic “surgical scrubbing” and donning of theatre attire in the operating theatre, with cursory adherence to standard IPC precautions in the wards:

For junior surgeons, there's a huge emphasis on what you do in the operating theatre - scrubbing, gowning and gloving - it's just *de rigueur.* The operating theatre occupies so much of their time it makes the ward stuff less worthy of their attention. But I'm sure most of the damage from [poor] infection control is done in the wards. (MD2)

### Leadership and role modelling

Leadership’s all about saying, doing and expecting others to do the right thing [MD3].

Participants emphasised the importance of leadership in moulding the IPC practices of junior doctors, who are likely to emulate those of senior consultants. They characterised *good* IPC leaders as consistently adhering to good practices, themselves, and being willing to discuss IPC principles and review practices and outcomes with their teams (see MD4’s anecdote about registrar hand hygiene audits, above, and Unit X director’s response to his patients’ HAIs below). Of course, clinical leadership is about much more than IPC practices:

I often talk about (a particular surgical unit) because they are a high performing team and their leadership is very strong across the board in all sorts of areas; they’re very solid as a team and the interns know the rules and abide by them even when (the consultants) are not there. (ND1)

On the other hand, bad or absent leadership is often also associated with poor IPC practice. If the “boss” ignores or disparages IPC practices, junior doctors would follow suit.

It’s about leadership and followership. Senior staff’s lack of role modelling - automatically the followers see that “it’s not a big deal for Sir, so it’s not a big deal for me”. (MD1)

One participant wondered whether leadership and role modelling might be less critical, now that junior doctors were increasingly aware of the importance of IPC and could influence consultants’ IPC behaviours, by reminding them of the policies. The more prevalent view was that the (still) powerful medical hierarchy was a strong deterrent against any attempt to correct the “boss”, even implicitly, for fear of retribution.

Where a junior person speaks to a more senior person (about) a protocol that hasn’t been performed correctly, the senior person feels threatened. The normal thing is to stamp your authority and say “well hang on, who are you?”. (MC2)

Most participants felt that any good habits learnt in medical school were likely to be superseded by hidden curricula assimilated during postgraduate training. Doctor participants admitted that they would not personally feel comfortable speaking to senior colleagues about poor IPC practices; on the other hand, some nurse participants said they would willingly speak up to a consultant or team with whom they had a good working relationship.

### Doctors’ accountability for healthcare-associated infections

Participants acknowledged the devastating effects, on patients, of serious HAIs, but pointed out that most doctors personally manage such complications infrequently and hear about only the worst cases at morbidity and mortality (M&M) meetings. Many doctors have little appreciation of the incidence, or impact on patients, of more minor infections. Moreover, HAIs are often regarded as unavoidable; they are rarely attributable to specific actions, omissions or even patterns of behaviour, because of the inevitable delay and multiple factors and people involved.

Nevertheless, participants described doctors’ contrasting reactions to their own patients’ HAIs: one saw it as a disaster, for which he felt personally responsible, another as a driver for change.

A nurse manager described starkly contrasting attitudes between two unit directors:

(Unit X’s director says) “What did we do wrong: what are we going to do to make sure this doesn't happen (again)?” He doesn't want to see bad things happen to patients.

(Unit Y’s director at the departmental M&M meeting) … . it's: “What's the patient done wrong this time? It's the patient's fault. It's not our fault. The patient's dirty (or) too fat; they should have looked after themselves better”. There's no accountability, no insight. (NM3)

Several participants noted that HAIs are often overlooked because of inadequate patient follow-up; they were highly critical of some senior consultants’ infrequent ward rounds, expectation that trainees would manage and follow-up patients, often with minimal supervision, and apparent lack of interest in patient outcomes.

Somebody who swans in, looks at something, swans out again and never understands that what he actually did was screwed up the patient’s life - that’s the problem. Interviewer: What can you do about it? Participant: Throw their noses in the data. (MD8)

Unfortunately, for most departments, very limited data are available. Collection of unit-specific HAI data was seen as the organisation’s, not individual unit’s, responsibility. While participants noted that monthly hand hygiene audit data were posted on ward notice boards, they believed that medical teams rarely reviewed them or, if they did, were skeptical about their accuracy and unaware of how they compared with those of other wards. One medical director (MD8) acknowledged that ward compliance data showed large discrepancies between wards and the lowest compliance rates amongst doctors, which were masked by the hospitals’ satisfactory results, overall. Another director (MD5) suggested that audit results overestimate actual compliance because auditors were not strict enough, but he rationalised doctors’ relatively low rates as being due to the fact that the auditors were nurses.

### Consultants’ professional obligations

In answer to an open question about general issues of concern, all medical divisional directors mentioned their frustration at some senior consultants’ apparent lack of commitment to their public hospital responsibilities, because of the demands or attractions of private practice.

[They] ... come in, do an operation, bugger off. They probably won't ever see that [public] patient again; they don't know what the outcome is. They don't have to front these patients in their office. [MD2]

Participants attributed this to better remuneration, working conditions, infrastructure and capital equipment in the private sector. While noting that VMOs value the prestige and challenging case-mix of a public hospital appointment, one director (MD2) pointed out that their contracts do not stipulate specific obligations (that many would take for granted), such as teaching and supervision of junior staff or accountability for patient outcomes. He suggested that, rather than relying on VMOs’ “altruism”, the public system should provide more attractive conditions.

If you want the talent you've got pay for it - an adequate salary, medical indemnity, secretary, reasonable office, parking space. It's a very big package, but that's what you need to do. If you offered them that they would be prepared to take a 30 or 40 per cent cut in income to concentrate their activities in one place and give up all the angst of being an employer. (MD2)

While this solution seemed unlikely in the current public health system, a senior manager mentioned the hospital executive’s plan to revise VMO contracts to define the “rules of engagement”, to which applicants must agree, including regular attendance at ward rounds and teaching sessions, trainee supervision and formal delegation of decision-making at weekends.

In common with many Australian public hospitals, the case hospital’s recent devolution of accountability for key performance indicators (KPIs), to divisional directors, had made them more aware and less tolerant of consultants, whom they perceived to be failing their implicit obligations, with impunity. In the context of this study, they linked this behaviour to ignorance of IPC policies and poor practice. However, they all agreed that instigating disciplinary action against senior doctors, in these circumstances, would be professionally and legally difficult, divisive and bitterly opposed, on principle, by medical organisations and most doctors, including those whose own behavior was above reproach. None of the divisional directors could offer solutions, other than more explicit employment contracts and/or more pay, but they conceded that it would still be difficult to monitor or enforce compliance. Despite their frustration, some directors described recent success, in delegating responsibility for clinical and administrative improvements, including in IPC, to unit directors, and assimilation of autonomous senior consultants into unit teams.

## Discussion

While doctors’ relatively poor IPC practices, overall, have been well-documented, this qualitative study is one of few which have deeply interrogated why this is so. Participants regarded senior doctors’ perceived entitlement to professional independence as a major contributor to how they choose to practice IPC and just one expression of how they fulfil implicit obligations to provide evidence-based patient care and clinical leadership. There was consensus that, although doctors are aware of the importance of IPC, for many it is not their highest priority. Nevertheless, most observe IPC policies – sometimes with modification - and/or do not object to being reminded. Uncertainty about the efficacy of some IPC measures and a lack of data about HAIs are barriers to doctors’ becoming more involved in IPC policy development and implementation. However, for most participants, the greatest concern was some senior consultants’ hostile disregard for IPC policies and its adverse effects on patients and other staff and their apparent immunity from censure.

### Autonomy

Participants regarded doctors as generally resistant to external rules, which they see as a challenge to their clinical autonomy and self-perception as independent thinkers [36]. In relation to IPC they may exercise autonomy, either by taking it very seriously (exemplary practice) or by choosing to remain ignorant or dismissive of basic IPC precautions, which participants interpreted as evidence of a perceived entitlement to decide, for themselves, what is important. It has been shown, previously, that compliance with hand hygiene is inversely related to educational level and seniority [37]. In making sense of this observation, we noted that many senior consultants completed their training and developed habituated behaviours at a time there was little focus on IPC, because of a prevalent belief that infectious diseases had largely disappeared [38]. While this belief is now known to have been misguided, the fact that it persists could be interpreted as a failure of continuing professional education (CPE).

Although CPE is a condition of annual medical registration, in Australia, it is largely confined to specialty topics. Specialist colleges endorse, and expect members to comply with, IPC guidelines and online IPC training is mandatory for all hospital staff, but many senior doctors choose to ignore both, with impunity. College expectations and hospital requirements are futile if professional autonomy is interpreted as meaning that compliance is discretionary [39]. Previous studies have also suggested that doctors’ poor compliance with guidelines, in general, reflects ignorance or skepticism about their effectiveness and/or an exaggerated confidence in their own judgment [40, 41]. As Charani et al suggest: *Senior doctors consider themselves exempt from following policy and practice, within a culture of perceived autonomous decision-making that relies more on personal knowledge and experience than formal policy* [42].

Senior consultants for whom IPC is a high priority may, nevertheless, seek to selectively ‘modify’ hospital IPC policies. This observation is supported by a recent study [43] that found that violations of transmission-based precautions were often not due to ignorance, but a clinicians’ judgment that the risks did not justify the extra staff time and cost or adverse effects on patients [44]. Participants gave examples of such modifications, which they regarded as sometimes appropriate. However, they are likely to be interpreted as arbitrary and a source of confusion and conflict; they might be avoided if doctors were more willing to be involved in the development and local implementation of IPC policies, as leaders or champions [45, 46].

### Leadership

Doctors’ apparent independence may be partly illusionary, if their clinical practice is guided by habituated behaviours, learnt during their early postgraduate training, by emulating teachers and supervisors, whom they admire or fear [47]. Participants identified leadership, as others have done [46], as an important determinant of (good or bad) IPC practice. Within a hierarchical hospital system, leadership is usually based on seniority, status and power [48]. Most senior consultants exercise their leadership roles appropriately and model acceptable or exemplary IPC practices but the attitudes of the minority, who repeatedly ignore or dispute basic IPC precautions, will also be transferred to junior doctors. Medical students are now taught about the importance of IPC and the risks of HAI and AMR but, as postgraduate trainees, they absorb the hidden curricula [47] of the specialties and units in which they train, which may be at odds with what they have been taught. Once entrenched, senior doctors’ habituated behaviours are difficult to change, since even their peers are deterred, by professional etiquette and their own uncertainties, from challenging colleagues’ unsafe practices [49].

### Accountability

Even doctors who are knowledgeable about and aware of the importance of IPC may struggle to understand how HAIs occur, because the effects of practice breaches are delayed, hidden and uncertain. Some regard HAIs as unavoidable or someone else’s fault – “the problem of many hands” [50] - and many have little concept of patients’ fears or the significant personal cost of even minor HAIs [51]. These problems are compounded by lack of data. Results of mandatory reporting to health authorities or internal incident reports of serious, but rare HAI-related events, such as bloodstream infections or unplanned readmissions, have little resonance and there are virtually no data about less serious HAIs. So it is impossible to monitor trends or reflect on individual consultant’s or unit’s performances *vis-à-vis* their peers, despite evidence that surveillance and feedback of results can motivate IPC improvement and reduce HAI rates [7, 52]. The lack of consistent national HAI surveillance, in Australia, is a recognised barrier to sustained IPC behavior change, particularly among doctors, and State- or hospital-based surveillance is highly variable [53].

### Professional obligations

The perceived failure of a small proportion of consultants to meet their obligations to the public hospital system was the issue that our participants spoke about most vehemently. Our participants felt strongly that senior doctors’ infrequent presence in the hospital and poor trainee supervision, (public) patient follow-up and IPC practices were not valid expressions of professional autonomy and agency, but manifestations of disrespect both for patients, who are exposed to unnecessary risk and colleagues who conform with, or are responsible for maintaining, IPC standards. Moreover, the hostile reactions of some doctors, to being reminded about basic IPC precautions, were interpreted as a manifestation of the bullying, which is endemic in healthcare settings in many countries, including Australia [54, 55], and as ‘yet another’ failure of professional self-regulation [56]. In this regard, recent media scrutiny of withdrawal of postgraduate training accreditation from two major Sydney public hospitals, largely as a consequence of bullying and harassment by senior medical staff [57, 58], raises the question of whether hospital administrators and specialist colleges will act to address *cultural* problems in medicine, including bullying, more effectively.

### Where to from here?

The Vanderbilt University Medical Centre’s system of co-worker observation and reporting is one promising approach to doctors’ (and others’) unprofessional behaviours. It involves, as a first step, a suitably trained colleague initiating an informal, respectful conversation with a doctor whose behaviour has been the subject of complaint from a patient or co-worker. In most cases this is apparently sufficient but, if a pattern of behaviour emerges, it is followed by staged escalation [59]. It has proven to be feasible and effective in reducing bullying [60], patient complaints [61] and poor IPC practices [62] and limits the need for more direct disciplinary action. It has been introduced or considered by several Australian hospitals [63] and the Royal Australasian College of Surgeons [64]. Such an holistic approach to organisation-wide culture change, would complement better surveillance and feed-back of HAI-related data and innovative strategies, such as the use of video-reflexive methods that have been used successfully to raise clinician awareness and improve IPC practices [65].

### Strengths and limitations

A major strength of this study was the seniority, varied clinical and management experience and broad vision of our participants, who were among the hospital’s most senior clinicians and clinician-managers/directors. Many of them were responsible for service quality and efficiency across multiple departments and acutely aware of the issues discussed. In addition, we believe that, because the interviewer was an “insider”, with comparable seniority and professional background, participants shared their insights and frustrations more candidly than they may have done with an external researcher. As researchers, we were inevitably influenced by our own perspectives and biases, which we sought to mitigate by discussion and self-reflection and by consulting as broad as possible a range of participants. As typical of qualitative research, they were but a small sample of the larger cohort of senior hospital clinicians, but represented a wide range of specialties, seniority, management responsibilities and attitudes to IPC.

The fact that participants and researchers were staff of a single hospital is appropriate for a case study but could also be seen as a limitation. However, our observations, experience and published literature suggest that the issues identified are common to most large, tertiary hospitals in Australia and countries with similar mixed public/private health systems. Moreover, limiting the study to a single site meant that all participants were familiar with the organisation’s idiosyncrasies and illustrative examples of doctors’ attitudes or practices were corroborated, and given added cogency, by multiple participants.

While participants expressed their opinions, frankly, about the attitudes and behaviours of colleagues, they generally did not identify them except by specialty. We did not seek to corroborate opinions about IPC practices with objective evidence of compliance, which was not the focus of this study, and none of the few units mentioned by participants, as having poor IPC practices, was represented amongst participants. This was unintended but may be seen as a limitation.

## Conclusions

Clearly many factors contribute to doctors’ IPC practices, other than (and sometimes contrary to) the norms, values and precepts of the medical profession. One of the most salient is how they interpret professional autonomy, which is a strongly defended principle of medical professionalism, but surely not intended to imply that doctors are entitled to ignore the policies of their employer organisations. Although participants sometimes defended doctors’ objections to, or ignorance of IPC “rules”, they clearly regarded doctors who reacted to reminders or requests to comply aggressively or unprofessionally, as an “out-group”.

In common with other preventive programs, IPC is sometimes a victim of its own (partial) success; there is a widespread but unsubstantiated impression that IPC practices are “good enough” and further improvements not cost-effective. However, the continuing prevalence of preventable HAIs and nosocomial transmission of MDROs and the threat of emerging infections mean that sustained improvement is needed. We argue that this will not be achieved without the full support and participation of all healthcare professions, including – perhaps especially – doctors. However, our findings suggest that improving doctors’ IPC practices will require greater commitment from professional organisations and healthcare administrators to a) more appropriately measure, and inform clinicians about, the adverse effects for patients of non-adherence and b) effectively enforce appropriate policies and practices that prevent these harms. The poor IPC practices of some doctors are just one aspect of the broader issue of unprofessional behaviour and immunity from criticism that must be addressed, if the medical profession, in general, is to be seen to conform with its espoused professional values and those of the community that supports it.

## Abbreviations

AMR: Antimicrobial resistance
HAI: Healthcare-associated infection
IPC: Infection prevention and control
M & M: Morbidity and mortality
MC/MC: Medical director/medical consultant
MDRO: Multidrug resistant organism
NC/ND/NM: Nursing consultant/nursing director/nursing manager
SAB: *Staphylococcus aureus* bacteraemia
SS: Staff specialist
VMO: Visiting medical officer
